# Alpha-linolenic acid supplementation effect in endoplasmic reticulum stress and adiponectin in abdominal subcutaneous adipose tissue in patients with type 2 diabetes mellitus

**DOI:** 10.1186/1758-5996-7-S1-A211

**Published:** 2015-11-11

**Authors:** Wallace Rodrigues de Holanda Miranda, Patrícia Moreira Gomes, Rebeca Antunes Beraldo, Milton Cesar Foss, Maria Cristina Foss-Freitas

**Affiliations:** 1 UFPI, Teresina, Brazil

## Background

In recent decades, it has seen a pandemic of type 2 Diabetes Mellitus (T2D) with high morbidity and mortality. T2D is associated with reduced levels of adiponectin and activation of endoplasmic reticulum stress (ERS), signals of chronic inflammation. In this context, it is necessary the study of new possibilities to improving this inflammation and there are many studies showing the anti-inflammatory effect of n-3 polyunsaturated fatty acid (PUFA).

## Objective

In this study we aimed to evaluate the effect of alpha-linolenic acid (ALA), a type of PUFA, supplementation in T2D patients on the molecular expression of adiponectin and ERS genes in abdominal subcutaneous adipose tissue (SAT).

## Materials and methods

We performed a placebo-controlled study, in a double-blind design with 20 patients with T2D, they received randomly 3g/day of ALA or placebo for 60 days and the SAT was collected by fine-needle aspiration before and after the supplementation. We evaluated the molecular expression of genes of adiponectin and ERS genes by real-time PCR and western blotting.

## Results

We observed the genic expression of adiponectin was increased after supplementation of ALA almost 90%, however we did not observe change of protein concentration by western blotting. In the ERS genes, we observed the reduction of the genic expression in XBP1 (20%), sXBP1 (70%) and increase in GRP78 (150%) and confirmed in protein concentration in the SAT. Furthermore we observed reduction in genic expression in IL-6 (80%) and IRS-1 (6-%), but it did not observed in protein concentration.

## Conclusion

Therefore ALA may modulate the ERS by the pathway of IRE1/XBP leading to increase the chaperones (BIP/GRP78), beside may modulate the adiponectin genic expression, but without change in protein concentration in SAT.

